# Influence of sucrose substitutes and agglomeration on volatile compounds in powdered cocoa beverages

**DOI:** 10.1007/s13197-019-04067-z

**Published:** 2019-09-05

**Authors:** Jolanta Kowalska, Hanna Kowalska, Beata Cieślak, Ewa Majewska, Marta Ciecierska, Dorota Derewiaka, Andrzej Lenart

**Affiliations:** 1 Department of Biotechnology, Microbiology and Food Evaluation, Warsaw University of Life Sciences, Faculty of Food Sciences, 159c Nowoursynowska St., 02-776 Warsaw, Poland; 2 Department of Food Engineering and Process Management, Warsaw University of Life Sciences, Faculty of Food Sciences, 159c Nowoursynowska St., 02-776 Warsaw, Poland

**Keywords:** Powdered cocoa beverage, Volatile ingredients, Agglomeration, Chromatographic analysis

## Abstract

Volatile aromatic substances are the main factors contributing to the acceptability of cocoa products. The beneficial effect of fat-free ingredients of cocoa beans on human health has been scientifically proven. This encourages the consumption of cocoa products as well as further research on improving their processing technology. The aim of this study was to analyse changes in the composition of volatile compounds and their impact on the sensory characteristics of an agglomerated cocoa powder mixture with modified composition for the raw material. The basic mixture was composed of 20% cocoa and 80% sucrose. Changes in mixture composition involved partial or total replacement of sucrose with maltodextrin or a mixture of glucose and fructose. Mixing and agglomeration were carried out in a fluid bed agglomerator. The analysis of volatile compounds was carried out using a gas chromatograph coupled with mass spectrometer, and 1,2-dichlorobenzene was used as an internal standard. The analysis showed the presence of over 70 various chemical compounds. Such volatile compounds as acetic acid, 2,3-butanediol, nonanal, and pentanoic acid, were found in almost all tested products. The highest content of acetic acid was determined in cocoa powder. In the case of the investigated cocoa beverages, the raw material composition and agglomeration affected their volatile compounds content. The analyses demonstrated a reduction in the content of volatile compounds caused by agglomeration.

## Introduction

A number of volatile compounds with the desired flavour and aroma notes, but also compounds with unpleasant notes are formed in the processing of cocoa beans, especially during fermentation and drying (Kongor et al. 2016). The presence of volatile compounds in processed cocoa beans products is a very important factor in determining consumer preferences. Approximately 400–600 various volatile compounds were identified in cocoa beans, belonging to approximately 20 different groups of chemical compounds. This makes the fragrance composition of cocoa very complex. Cocoa beans have delicate taste and aroma depending on their place of origin and on climatic and soil conditions that are characteristic for that particular place. The properties of cocoa beans influence the quality of cocoa bean products (Crafack et al. 2014; Álvarez et al. 2016; Kongor et al. 2016).

Compounds ensuring the characteristic aroma are valuable ingredients of cocoa beans. They constitute a highly complex group and are composed of fractions of volatile and non-volatile compounds. Important aromatic substances present in cocoa beans are hardly volatile. Those are mainly aromatic acids. Moderately volatile compounds, containing residual pyrazine, thermally-processed in high humidity conditions, form furyl compounds and linalool and impart a floral taste and roasted beans aroma. Volatile aldehydes are formed upon decomposition of amino acids, loss of which improves the taste of the product. Aroma is also developed by non-volatile components of cocoa beans, including flavones, amino acids, tannic acids, carbohydrates, and purines. The majority of aroma-developing compounds are formed during fermentation and thermal processes, mostly by the drying and roasting of beans, as well as alkalisation. The type and quantity of individual aromatic compounds depend on the chemical composition and structure of the product, availability of precursors of those compounds, temperature and time of heating, and water activity (Afoakwa et al. 2012; Álvarez et al. 2016; Hu et al. 2016). The abundance of natural oxidizers present in cocoa beans and in cocoa powder, should favour their regular consumption (EFSA 2012).

Cocoa powder is widely used in the confectionery and cake industry, and in the cosmetic industry. The use of cocoa powder to prepare a beverage is a difficult task, because of its poor wettability and solubility. The combination of powdered cocoa and other components, including starch, sugar, and milk powder, facilitates its solubility and leads to the development of a cocoa product with additives (referred to as a cocoa beverage) (Kowalska and Lenart 2005; Benković and Bauman 2011; Kowalska et al. 2011). These beverages are readily soluble in milk or water. A typical cocoa beverage is a mixture of cocoa powder (approximately 20%) and sucrose (approximately 80%), with an addition of lecithin and enriched with vitamins. One of the methods of cocoa manufacturing with additives is mixing appropriate proportions of cocoa powder and other ingredients. There are also some more complex methods of obtaining cocoa beverages. Such methods as agglomeration and coating allow manufacturing an instant product with specific reconstitution properties (Vissotto et al. 2014; Ostrowska-Ligęza and Lenart 2015).

Powdered cocoa beverage in the form of a mixture of cocoa with other components, pursuant to Directive 2000/36/EC referred to as “sweetened cocoa powder”, is characterised by a high caloric value (approximately 370–400 kcal/100 g) (Directive 2000/36/EC). Also its glycaemic index is high, usually over 60. As instant cocoa is consumed mostly by children, the development of a product with modified raw material composition is recommended involving either total or partial replacement of sucrose with another saccharide, for example, a mixture of glucose and fructose, or maltodextrin, in order to preserve the expected sweet taste (Brand-Miller et al. 2013; Lee et al. 2013). Considering that the intensity of the sweet taste of fructose is higher and of glucose lower compared to sucrose, the quantity of those compounds added to achieve a similar taste effect may be reduced, and as a result, the caloricity of the resulting product will be lower.

On the other hand, maltodextrin is characterised by low sweetness, but similar to glucose; the compound promotes perception of sweet taste and intensifies product taste and aroma (Moskowitz 1970; Grembecka 2015).

The aroma and taste of cocoa powder are very intensive and characteristic. Therefore, the presence of cocoa powder in a mixture influences the feeling of powdered aroma and taste of cocoa beverage. The colour of the final product is important for its reception by a consumer. Thus, a dark colour suggests strong taste and aroma, and a lighter colour is associated with milder aroma (Miller et al. 2008). Darker products are alkalised, and their taste and aroma are fixed during the process. This makes them more perceptible. Alkalisation influences not only the colour, taste, and aroma of cocoa powder, but also its pH. Acidity and alkalinity also affect taste perception and the composition of aroma compounds. The pH of cocoa powder with intensive red-brown colour and characteristic chocolate aroma ranges between 7.4 and 8.0 (Li et al. 2014; Taş and Gökmen 2016).

The process of manufacture of instant cocoa involves a broad spectrum of chemical reactions, which is due to a complex matrix. For a consumer, the organoleptic properties influencing the quality of a product include: the aroma (associated with the presence of volatile compounds), the taste (determined by the quantity and type of sugars contained in the product), and the volume of water (determining the solubility and stability of the product). The composition of cocoa powder and the parameters of its processing have a strong influence on volatile compounds presence after processing. The type and quantity of volatile compounds may, therefore, be indicators of product quality.

The aim of this study was to analyse changes in the composition of volatile compounds and their impact on the sensory characteristics of an agglomerated cocoa powder mixture with modified raw material composition.

## Materials and methods

### Materials

Raw materials were powders with the trade names: low-fat cocoa powder, sucrose (instant), medium-saccharified maltodextrin, glucose and fructose. Cocoa powder and sucrose were bought from a manufacturer of powdered cocoa beverages, maltodextrin was obtained from a supplier of additives for the food industry, and glucose and fructose were procured at a retail point so-called “healthy food” shop. The basic mixture contains 20% cocoa and 80% sugar. To basic mixture 0.5% lecithin is added. The most important is ratio 2:8 between cocoa and sugar in basic mixture. Changes in mixture composition involved partial or total replacement of sucrose with maltodextrin or a mixture of glucose and fructose. The cocoa powder percentage was always 20% (Table 1).

**Table 1:** Codes of samples: raw materials, mixtures

Product name	Codes of samples	
	Powders	Agglomerates
*Raw materials*		
Cocoa	P1	A1
Sucrose	P2	A2
Maltodextrin	P3	A3
Glucose	P4	A4
Fructose	P5	A5
*Mixtures*		
20% cocoa + 80% sucrose	P6	A6
20% cocoa + 40% sucrose + 40% glucose and fructose	P7	A7
20% cocoa + 40% sucrose + 40% maltodextrin	P8	A8
20% cocoa + 40% maltodextrin + 40% glucose and fructose	P9	A9
20% cocoa + 80% glucose and fructose	P10	A10

*P* non-agglomerated (powders), *A* agglomerated and dried at 68 °C for 15 min (agglomerates)

All raw materials, mixtures and agglomerates were stored in tightly closed plastic containers, in a dark room at room temperature, until analysed.

### Preparation of powder mixtures

The technology of obtaining agglomerates of raw materials and mixtures consisted in mixing the components (0.3 kg) in a tank of the STREA 1 (Niro-Aeromatic AG) agglomerator for 2 min. The powder was sprayed by water at an optimal flow rate of 6 × 10^−3^ kg/s for 15 min and the air pressure in the spraying nozzle was 0.2 MPa. After the end of wetting, the agglomerate was dried at 68 °C for 15 min (thermal treatment).

The obtained agglomerates were granulometrically analysed on sieves with mesh size ranging from 0.0 to > 2.0 mm. Fractions with particle size of 0.2–2.0 mm were used for volatile compounds testing (Kowalska et al. 2011).

### Volatile compounds analysis

The most favourable conditions of volatile compounds analysis were determined experimentally because of the complexity of the matrix (mixtures and agglomerates) and considering thermal operations (drying at 68 °C for 15 min) used to prepare the samples.

Based on the chromatographic analysis, the most favourable results were obtained following the application of the fibre DVB/CAR/PDMS (divinylbenzene/carboxene/polydimethylsiloxane) 50/30 μm, extraction at 40 °C, for 30 min.

Based on results of preliminary studies, 1,2-dichlorobenzene was selected as an internal standard (IS) and used in the quantity of 1.3 ppb.

Approximately 1 g of the examined product was weighed into a 10 mL glass vial with 1 μL of internal standard (0.01% 1,2-dichlorobenzene in methanol). The vial has been sealed. Extraction of volatile compounds was performed at 40 °C for 30 min, by means of SPME *(*Solid Phase Microextraction*)* (CAR/PDMS/DVB). The desorption was performed in the injector of a gas chromatograph coupled with a mass spectrometer (QP2010, Shimadzu) equipped with a ZB-WAX capillary column (30.0 m length, 0.25 mm internal diameter, 0.25 μm film thickness, Phenomenex). The parameters of the desorption were as follows: duration 2 min and temperature 190 °C. The injection was done in the split mode. The following parameters have been used for the identification of volatile compounds: helium flow rate—0.79 mL/min; oven temperature—40 °C for 2 min, increased at 6 °C/min to 190 °C (3 min); interface temperature—170 °C and ion source temperature—180 °C.

Mass acquisition range was from 40 to 350 m/z. Compound identification was based on comparison with reference spectra from libraries (Wiley7N2, NIST147). The so-called relative peak areas were calculated by dividing the peak area of every compound by the internal standard’s peak area. To increase the clarity of figures, presented data were standardized to 0 mean and 1 standard deviation (Zhu et al. 2016).

The compounds tested were identified and quantified and resources of libraries: 147, PAL 600, and SZTERP, were used to analyse results of chromatographic separation. Identified compounds were checked in all three available libraries, before they were placed on the list of results.

The analysis revealed the presence of over 70 various volatile compounds. Only those compounds whose content exceeded 0.5% of the peak area of all volatile compounds in the particular chromatogram were analysed. Values below that limit (0.5%) were recognised by comparison with spectra library, with precision below 80%. The identified volatile compounds belonged mainly to six different chemical groups: alcohols, acids and esters, aldehydes and ketones, and cyclic components including furans. The content of volatiles was calculated on the basis of the area under the peak, taking into account sample weight and expressed in µg*100 g^−1^ d.m.

### Sensory analysis

The quantitative descriptive analysis (QDA) was performed in order to create chocolate sensory profiles. The team was collected and trained; the following distinguishing factors were defined and characterised: flavour (chocolate, cocoa, acrid, acidic), taste (chocolate, cocoa, acidic), sweetness, bitterness, and astringency. The assessment was made using a ten-point scale, where “0” meant the lack of a given feature, not perceptible, while “10”—a perceptible, intense feature. The determination of the sensory profile was performed on disposable plates bearing appropriate three-digit codes, in three replications. Samples (mixtures and agglomerates) were served under white light in cabins at temperature controlled to  ± 20 °C. Water was provided for palate cleansing (ISO 13299:^2016^).

### Statistical analysis

The statistical analysis of volatile compounds was completed based on three determinations, following calculation of each test peak area in relation to IS. Volatiles’ mean relative peak areas were compared by means of ANOVA. All the calculations were performed in Statistica 12.0.

The correlation matrix was analysed to determine the significance of the impact of changes in raw material composition and the agglomeration process on the sensory attributes as well as the dependence between volatile compounds and sensory characteristics.

The principal component analysis (PCA) was carried out using Statistica 12.0, only for the products with the identified compounds. PCA analysis was performed to correlate the volatile compounds in the powdered cocoa beverage and their mixtures and the results of their sensory analysis.

## Results and discussion

**Fig. 1:**
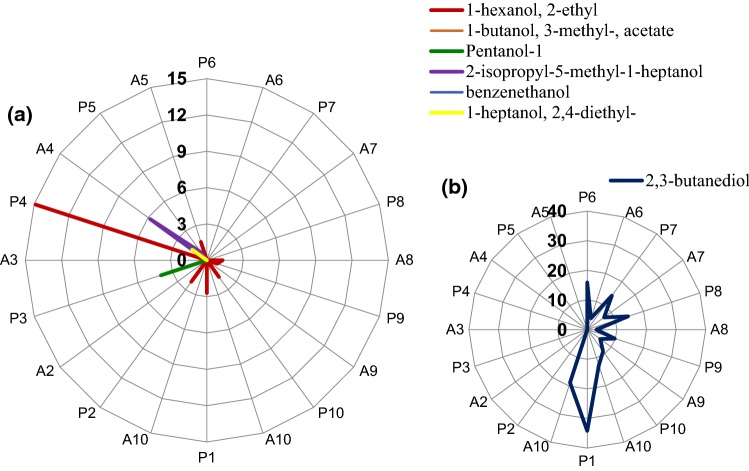
Volatile compounds belonging to the group of alcohols present in powders, mixtures and agglomerates of powdered cocoa beverages expressed in µg*100 g^−1^ d.m: **a** alcohols, **b** 2,3-6 ingredient. Codes of samples as in Table 1. *P* powder, *A* agglomerate

The presence of seven compounds belonging to the group of alcohols was detected in the tested products (Fig. 1).

2,3-Butanediol (Fig. 1) was present in cocoa powder (P1, A1; Table 1), its mixtures (P6, P7, P8, P9, P10; Table 1) and agglomerates (A6, A7, A8, A9, A10; Table 1). It was absent in the other analysed products (P2-A5; Table 1). The presence of 1-hexanol, 2-ethyl (Fig. 1) was found in cocoa powder (P1; Table 1), sucrose (P2; Table 1) and glucose (P4; Table 1), and its content decreased below 0.5% as a result of agglomeration (A1, A2, A4; Tab 1).

The compounds identified in cocoa powder obtained during the fermentation process were formed during drying and also as Maillard reaction products in the roasting process. As demonstrated by Aculey et al. (2010), a series of volatile compounds with high concentrations is formed depending on the duration of fermentation and drying of beans. After 1 day of beans fermentation, 2-methylpropanol, 2-pentanol, methyl acetate, 2-propylene were detected, and after 3 days of the process—propionic acid, acetic acid, benzaldehyde, linalool, and many others. The so-called industrial beans (Forastero—grown on a large scale) are subjected to long fermentation, resulting in the formation of larger amounts of volatile compounds. In addition, according to the study of Rodriguez-Campos et al. (2012), cocoa beans drying in the sun, in natural conditions, affect the increase in contents of their alcohols, esters or pyrazines. Numerous studies have shown an effect of high temperature on the content and quantity of volatile compounds (Rodriguez-Campos et al. 2012; Álvarez et al. 2016; Kongor et al. 2016).

The content of alcoholic compounds decreased during agglomeration. The analysis of the identification volatile compounds demonstrated an effect of raw material composition change, consisting in partial or total replacement of sucrose with another sugar, on their contents.

The content of 2,3-butanediol was the highest (Fig. 1) in the cocoa powder (P1; Table 1), while less than 50% of the determined 2,3-butanediol was observed in mixtures. Volatile compounds content in the tested mixtures was also affected by changes in mixtures composition. Partial replacement of sucrose with another sugar caused a decrease in 2,3-butanediol content (Fig. 1) by approximately 10% (P7, P8; Table 1), whereas complete elimination of sucrose in the composition caused a decrease by over 40% (P9, P10; Table 1) (Fig. 1).

**Fig. 2:**
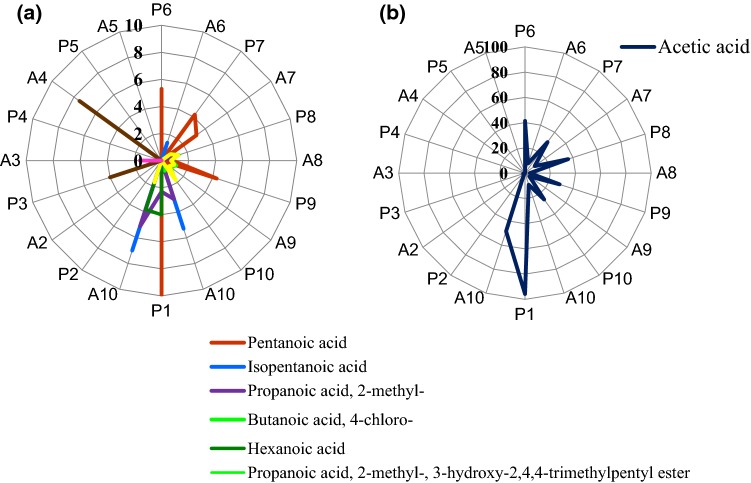
Volatile compounds belonging to the group of acids and esters present in powders, mixtures and agglomerates of the powdered cocoa beverages expressed in µg*100 g^−1^ d.m: **a** acids and esters present, **b** acetic acid. Codes of samples as in Table 1. *P* powder, *A* agglomerate

Another ten compounds identified belonged to the group of acids and esters (Fig. 2). Acetic acid dominated in that group and among all determined compounds; it was detected in 13 of the 20 tested samples. The highest acetic acid content was determined in cocoa powder (P1; Table 1), and is associated with the cocoa beans fermentation process, being a part of the manufacturing process (Rodriguez-Campos et al. 2012; Álvarez et al. 2016). Acetic acid was also found in mixtures P6, P7, P8, P9, and P10 (Table 1). Its content was over two or three times lower than in the raw material, which is due to the percentage of cocoa powder (20%) in mixtures composition. The agglomeration process contributed to a decrease in acetic acid content (Fig. 2, Table 1) compared to mixture of the same composition (approximately 3–5-fold decrease) (A6, A7, A8, A9, A10; Table 1). No clear effect was observed of composition modification on the content of the determined acid.

Cocoa powder (P1; Table 1) was found to contain: acetic acid, pentanoic acid, propanoic acid, 2-methyl-and hexanoic acid (Fig. 2). The latter compound was also present in maltodextrin. Isopropyl myristate was detected in maltodextrin following agglomeration, but not in the other samples.

Pentanoic acid (Fig. 2) was present in most of the mixtures (P6, P7, P9; Table 1), however, its content decreased (Fig. 2) after the agglomeration process (A6, A7, A9; Table 1). Its highest content was determined in cocoa powder.

The products marked as A7–P10 (Table 1) were found to contain propanoic acid, 2-methyl-, 3-hydroxy-2,4,4 trimethylpentyl and propanoic acid, and 2-methyl-2,2-dimethyl-1-(2-hydroxy-1-methylethyl) propyl (Fig. 2). The content of acids in fermented cocoa beans was higher than that of the other groups of volatile compounds. The increase in acids content is due to the metabolism of sugars, among others these contained in cocoa liquor (Frauendorfer and Schieberle 2008; Owusu et al. 2012). Some loss of acids was noted as a result of high-temperature processing during drying or roasting; it ranged from 10 to 40% depending on process parameters, including time and temperature of the heating factor applied during the beans roasting process (Saltini et al. 2013; Kongor et al. 2016). Nevertheless, some of the acids remain in the beans and are present in the products of the bean processing. Completed analyses demonstrated differences in contents of the majority of determined acids in the samples with modified raw material composition. The above mentioned acetic acid, being a major compound in cocoa powder, and therefore also in mixtures, was present in a smaller amount in those products in which sucrose was replaced by another sugar. That supports the conclusions suggested by Rosicka-Kaczmarek (2006).

Some acids present in processed products of cocoa beans may cause unfavourable taste and smell sensations. However, some acids, e.g. hexanoic acid and octane with a sweet aroma, can cause a pleasant aroma of products (Menezes et al. 2016).

**Fig. 3:**
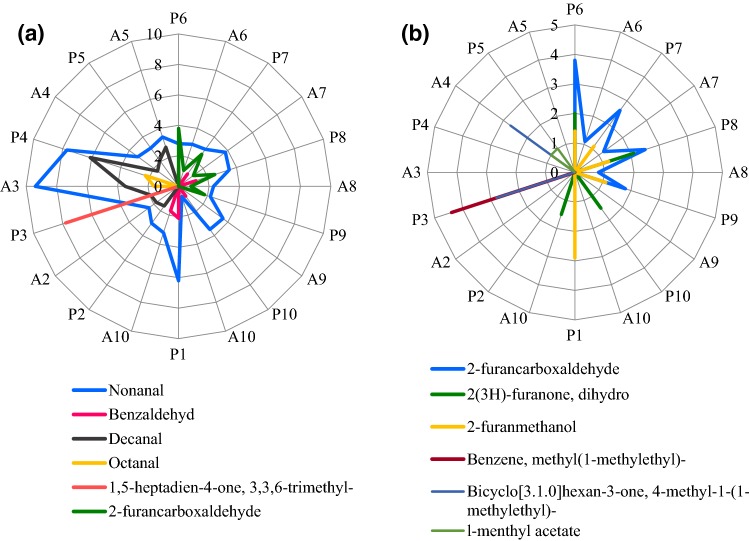
Volatile compounds belonging to the group of: **a** aldehydes and ketones, **b** furans and other cyclic compounds present in powders, mixtures and agglomerates of the powdered cocoa beverages expressed in µg*100 g^−1^ d.m. Codes of samples as in Table 1. *P* powder, *A* agglomerate

Six compounds belonging to the group of aldehydes and ketones were found in the analysed products (Fig. 3a). Nonanal was present in all analysed products, however its content decreased in most of the samples during the agglomeration process (Fig. 3a). The presence of benzaldehyde was found in cocoa powder (1; Table 1) and agglomerated cocoa (2; Table 1) as well as in most of the mixtures (P6, P7, P8, P9, P10; Table 1) (Fig. 3a). After the agglomeration process, benzaldehyde was absent in all the products.

Powder components, except cocoa (P1; Table 1), showed presence of decanal (Fig. 3a) whose highest content was determined in glucose (P4; Table 1), and the lowest in sucrose (P2; Table 1). The agglomeration process caused an increase in its content in raw materials from about 12% in sucrose (A2; Table 1) to about 45% in maltodextrin (A3; Table 1). An opposite correlation was demonstrated only for glucose (A4; Table 1). Decanal content decreased by over 3.5 times, as a result of temperature and time of agglomeration (Fig. 3a).

2-Furancarboxaldehyde (Fig. 3a) was detected in samples marked P6 to P9 (Table 1), while it was absent in the other products studied. It is naturally found in a variety of foods and beverages, in particular fruit and vegetables, and may also be formed during the processing or cooking of many food products, in particular roasted nuts, coffee, wine, and cocoa beans.

The content of aldehyde compounds remained below the accepted threshold limit in all mixtures. Other compounds of that group occurred in single components.

The last analysed group was that of furans and other cyclic compounds (Fig. 3b), which were mostly found in mixtures and agglomerates. As a result of agglomeration, there was a decrease in contents of the majority of the analysed volatile compounds. Four compounds were determined as belonging to the group of furans and others cyclic compounds (Fig. 3b). 2-Furanmethanol was also present in the tested mixtures and in cocoa powder. Its content was lower than 0.5% only in the product P10 (Table 1). According to Aprotosoaie et al. (2016), furans are also important in the development of aroma and cocoa flavour in addition to compounds from the group of acids, aldehydes and ketones.

According to the studies confirmed by EFSA (2017), furans are compounds that pose human hazards with a potential for carcinogenic and cytogenic effects. Kowalski et al. (2008) explained that the mechanism of furan formation in food includes the thermal breakdown of sugars, such as glucose, fructose and lactose, and that furan can be formed by the transformation of amino acids (alanine, cysteine, casein), amino acid mixtures, carbohydrates, vitamins, polyunsaturated fatty acids (PUFA) and carotenoids. Alkyl derivatives of pyrazine have been recognised as crucial components of the aroma of roasted material. The content of pyrazine-type compounds included in the aroma of roasted cocoa beans depends on the type of beans (Wu et al. 2014).

2-Furanmethanol (Fig. 3b) was determined in cocoa powder (P1; Table 1) and in the majority of mixtures (P6, P7, P8, P9; Table 1). After the agglomeration, its content decreased below 0.5%. In contrast, 2(3H)-furanone, dihydro was determined in agglomerated cocoa powder (A1; Table 1), in the mixture with the basic composition (P6; Table 1) and mixtures P8 and P10 (Table 1), while it was absent in the other samples tested.

In most of the samples, the agglomeration and final drying (for 15 min at 68 °C) process caused reduced contents of individual volatile compounds. Only in some cases an increase in a volatile content was observed after agglomeration, compared to the mixture of the same composition (Fig. 2). That was the case of nonanal, when differences were over 20% and approximately 40%, respectively. According to results of the previous study, the higher temperature reduced contents of most of the volatile compounds (Rodriguez-Campos et al. 2012; Kongor et al. 2016).

The high-temperature processing was also reported to cause a decrease in bitterness and tartness characteristic of cocoa beans (Ribeiro et al. 2009). Nevertheless, with regard to the mixtures containing 20% cocoa powder, it is essential that the characteristic and desirable flavour notes are palpable and develop the characteristics of the cocoa product. Therefore, a reduction in the content of some volatile compounds under the influence of high temperature treatment may reduce the intensity of the perceptible cocoa notes and thus cause the lack of product acceptance by consumers (Rodriguez-Campos et al. 2012; Kongor et al. 2016). The process parameters should be optimised to enable preserving desirable and expected volatile compounds and minimising those that can contribute to undesirable sensory effects, e.g. some acids. This should be analysed taking into account the precursor compounds of these selected volatiles. Furan is formed mainly by thermal degradation of sugars and carbohydrates, and the oxidative degradation of proteins and sugars is the main route of aldehyde formation (Ribeiro et al. 2009).

**Fig. 4:**
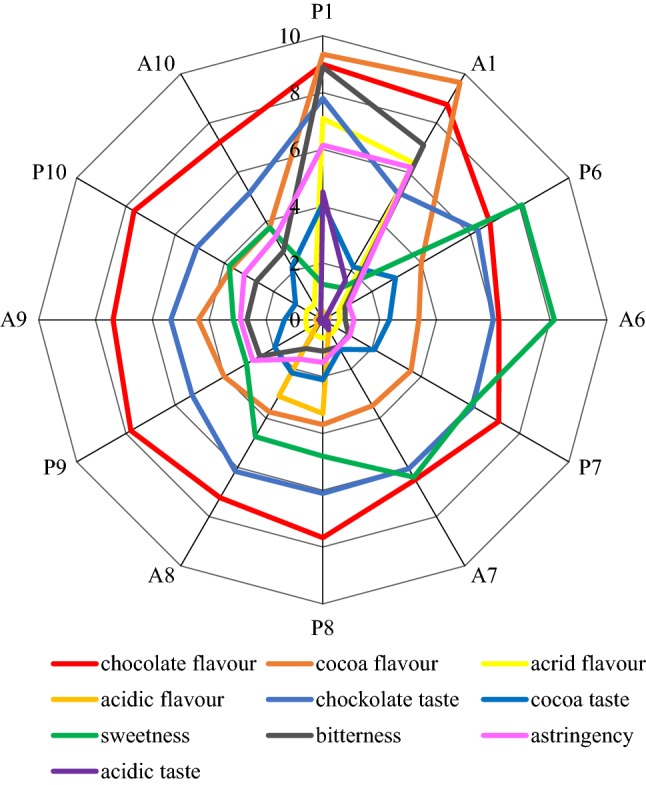
Sensory profiles of the cocoa powder, mixtures and agglomerates of the powdered cocoa beverages. Codes of samples as in Table 1. *P* powder, *A* agglomerate. Point scale 0–10

The cocoa powder (P1; Table 1) was evaluated for its sensory characteristics, such as cocoa and chocolate flavour and taste, astringency, tartness, acid taste or acrid flavour (Fig. 4). For samples containing 80% sucrose (P6, A6; Table 1), the experts of sensory evaluation defined as the most perceptible and characteristic, mainly in terms of taste and aroma. The combination of cocoa powder with sweeteners (saccharose, glucose, fructose, maltodextrin) allowed neutralizing bitterness, astringency, acid taste or biting aroma. The smallest differences were found in chocolate taste and aroma assessments, however, the taste and aroma of cocoa in the mixtures with sugars was much less perceptible.

A more noticeable cocoa taste (Fig. 4) was found in the samples containing sucrose (P6, A6, P7, A7, P8, A8; Table 1). On the other hand, according to the panellists, the mixtures and agglomerates with the basic composition (P6, A6; Table 1), i.e. with 20% cocoa and 80% sugar, were characterised by the least recoverable chocolate flavour. The agglomeration did not affect or slightly affected the change in the perceptibility of the analysed sensory attributes in most of the analysed samples.

The obtained results of the sensory evaluation indicate the formation of specific distinguishing factors of the foreign taste. A difference was demonstrated between the analysed mixtures with a modified composition of raw materials. For example, a foreign taste (Fig. 4) was determined in a product containing maltodextrin, which is characterised by specific taste qualities (A7, P8, A8; Table 1). Statistical inference was performed using the two-way ANOVA to identify homogeneous groups. The influence of cocoa powder on the sensory properties of mixtures, and the impact of raw materials and the agglomeration process on the studied sensory characteristics were analysed. A statistically significant correlation confirmed the effect of the modification of raw material composition on the examined quantity of the mixtures. However, the effect of agglomeration on the sensory characteristics of the finished product has not been demonstrated (Table 2). The correlation matrix was carried out to determine the correlation between volatile compounds and sensory variables. The significant correlation is marked in Table 3 with the letter and bold with the correlation value.

**Table 2:** Correlation matrix of the impact of raw material composition modification and agglomeration process on sensory characteristics of cocoa mixtures with additions

	P1	A1	P6	A6	P7	A7	P8	A8	P9	A9	P10	A10
P1	1.00	**0**.**93a**	0.09	0.04	0.23	0.19	0.13	0.14	0.57	0.59	0.52	0.51
A1	**0**.**93a**	1.00	0.16	0.12	0.29	0.25	0.22	0.23	0.62	**0**.**65a**	0.60	0.57
P6	0.09	0.16	1.00	**1.00a**	**0.97a**	**0.97a**	**0.86a**	**0.87a**	**0.77a**	**0.76a**	**0.79a**	**0.80a**
A6	0.04	0.12	**1.00a**	1.00	**0.96a**	**0.97a**	**0.84a**	**0.85a**	**0.74a**	**0.74a**	**0.77a**	**0.78a**
P7	0.23	0.29	**0.97a**	**0.96a**	1.00	**0.99a**	**0.92a**	**0.93a**	**0.88a**	**0.87a**	**0.88a**	**0.89a**
A7	0.19	0.25	**0.97a**	**0.97a**	**0.99a**	1.00	**0.90a**	**0.91a**	**0.84a**	**0.84a**	**0.86a**	**0.85a**
P8	0.13	0.22	**0.86a**	**0.84a**	**0.92a**	**0.90a**	1.00	**1.00a**	**0.85a**	**0.84a**	**0.84a**	**0.83a**
A8	0.14	0.23	**0.87a**	**0.85a**	**0.93a**	**0.91a**	**1.00a**	1.00	**0.85a**	**0.85a**	**0.85a**	**0.84a**
P9	0.57	0.62	**0.77a**	**0.74a**	**0.88a**	**0.84a**	**0.85a**	**0.85a**	1.00	**0.99a**	**0.99a**	**0.99a**
A9	0.59	**0.65a**	**0.76a**	**0.74a**	**0.87a**	**0.84a**	**0.84a**	**0.85a**	**0.99a**	1.00	**0.99a**	**0.98a**
P10	0.52	0.60	**0.79a**	**0.77a**	**0.88a**	**0.86a**	**0.84a**	**0.85a**	**0.99a**	**0.99a**	1.00	**0.99a**
A10	0.51	0.57	**0.80a**	**0.78a**	**0.89a**	**0.85a**	**0.83a**	**0.84a**	**0.99a**	**0.98a**	**0.99a**	1.00

Sensory attributes/factors	Addition of cocoa powder effect		Raw material composition effect		Agglomeration effect	
	*p* values	Homogeus groups	*p* values	Homogeus groups	*p* values	Homogeus groups
Acidic taste	0.00*	A	0.00*	B′	0.8767	a
Acidic flavor		A, B		B′, C′		a
Acrid flavour		A, B		A′, B′, C′		a
Cocoa taste		A, B		A′		a
Astringency		A, B, C		A′		a
Bitterness		B, C, D		A′, C′		a
Sweetness		C, D, E		D′		a
Cocoa flavour		D, E		E′		a
Chockolate taste		E, F		D′		a
Chocolate flavour		F		F′		a

The statistical data with letter “a”—were bolded and means statistically significant influence of the change in the composition of raw materials and the agglomeration process on the sensory factors

*Difference significant; A, B,… or A′, B′ … or a, b…—homogenous groups

**Table 3:** The matrix of the correlation of volatile compounds and the sensory characteristics of cocoa mixtures

	Acidic flavor	Chockolate taste	Cocoa taste	Sweetness	Bitterness	Astringency	Acidic taste	2,3-Butanediol	1-Hexanol, 2-ethyl	Nonanal	Benzaldehyde
Chocolate flavor	− 0.01	0.18	0.40	− **0.91a**	**0.88a**	**0.89a**	**0.72a**	**0.73a**	0.56	0.46	**0.77a**
Cocoa flavor	− 0.22	0.33	0.55	− **0.71a**	**0.95a**	**0.90a**	**0.85a**	**0.76a**	0.39	0.55	**0.72a**
Acrid flavor	− 0.21	0.42	**0.60a**	− **0.67a**	**0.95a**	**0.88a**	**0.90a**	**0.80a**	0.45	**0.60a**	**0.73a**
Acidic flavor	1.00	0.19	0.00	0.06	− 0.30	− 0.30	− 0.18	− 0.18	0.05	− 0.06	− 0.10
Chockolate taste	0.19	1.00	**0.79a**	0.07	0.30	0.07	**0.69a**	**0.61a**	0.49	**0.69a**	0.43
Cocoa taste	0.00	**0.79a**	1.00	− 0.12	0.52	0.37	**0.73a**	**0.77a**	0.42	0.39	0.61 **a**
Sweetness	0.06	0.07	− 0.12	1.00	− **0.82a**	− **0.89a**	− 0.57	− 0.48	− 0.49	− 0.28	− 0.45
Bitterness	− 0.30	0.30	0.52	− **0.82a**	1.00	**0.97a**	**0.89a**	**0.78a**	0.55	0.55	**0.67a**
Astringency	− 0.30	0.07	0.37	− **0.89a**	**0.97a**	1.00	0.75 **a**	**0.67a**	0.49	0.38	**0.59a**
Acidic taste	− 0.18	**0.69a**	**0.73a**	− 0.57	**0.89a**	**0.75a**	1.00	**0.86a**	**0.65a**	**0.75a**	**0.67a**
2,3-Butanediol	− 0.18	**0.61a**	**0.77a**	− 0.48	**0.78a**	**0.67a**	**0.86a**	1.00	0.46	**0.59a**	**0.87a**
1-Hexanol, 2-ethyl	0.05	0.49	0.42	− 0.49	0.55	0.49	**0.65a**	0.46	1.00	0.54	0.40
Nonanal	− 0.06	**0.69a**	0.39	− 0.28	0.55	0.38	**0.75a**	**0.59a**	0.54	1.00	0.55
Benzaldehyde	− 0.10	0.43	**0.61a**	− 0.45	**0.67a**	**0.59a**	**0.67a**	**0.87a**	0.40	0.55	1.00
Acetic acid	− 0.14	**0.64a**	**0.76a**	− 0.46	**0.77a**	**0.64a**	**0.86a**	**0.96a**	0.56	**0.69a**	**0.94a**
Pentanoic acid	− 0.26	**0.78a**	**0.71a**	− 0.10	0.41	0.22	**0.67a**	**0.70a**	0.52	0.57	**0.58a**
Isopentanoic acid	− 0.25	− 0.43	0.02	− 0.36	0.41	0.53	0.10	0.16	− 0.31	− 0.38	0.08
Propanoic acid, 2-methyl	− 0.13	− 0.08	0.31	− **0.62a**	**0.73a**	**0.79a**	0.50	0.56	− 0.01	0.02	0.46
Butanoic acid, 4-chloro	− 0.13	− 0.30	0.01	− 0.11	0.01	0.11	− 0.12	0.01	− 0.19	− **0.60a**	− 0.32
Hexanoic acid	− 0.21	0.40	**0.58a**	− **0.67a**	**0.95a**	**0.88a**	**0.89a**	**0.79a**	0.43	**0.58a**	**0.73a**
Propanoic acid, 2-methyl-, 3-hydroxy-2,4,4-trimethylpentyl ester	0.24	− 0.52	− **0.59a**	− 0.42	0.08	0.22	− 0.20	− 0.22	0.06	0.05	0.10
Propanoic acid, 2-methyl-,2,2-dimethyl-1-(2-hydroxy-1-methylethyl) propyl ester	0.21	− 0.52	− 0.56	− 0.43	0.13	0.27	− 0.15	− 0.17	0.07	0.05	0.16
2-Furancarboxaldehyde	0.20	0.21	0.15	**0.67a**	− 0.57	− **0.66a**	− 0.37	− 0.04	− 0.39	− 0.14	0.15
2(3H)-Furanone, dihydro	0.24	− 0.12	0.00	0.07	− 0.02	0.02	− 0.13	0.19	− 0.15	0.06	0.46
2-Furanmethanol	0.01	**0.79a**	**0.80a**	− 0.18	0.43	0.26	**0.67a**	**0.82a**	0.50	**0.59a**	**0.75a**

	Acetic acid	Pentanoic acid	Isopentanoic acid	Propanoic acid, 2-methyl-	Butanoic acid, 4-chloro-	Hexanoic acid	Propanoic acid, 2-methyl-, 3-hydroxy-2,4,4-trimethylpentyl ester	Propanoic acid, 2-methyl-,2,2-dimethyl-1-(2-hydroxy-1-methylethyl) propyl ester	2-Furancarboxaldehyde	2(3H)-furanone, dihydro	2-Furanmethanol
Chocolate flavor	**0.75a**	0.31	0.29	**0.65a**	− 0.08	**0.81a**	0.34	0.37	− 0.41	0.18	0.47
Cocoa flavor	**0.77a**	0.38	0.46	**0.77a**	− 0.14	**0.99a**	0.09	0.15	− 0.42	0.10	0.40
Acrid flavor	**0.81a**	0.43	0.42	**0.74a**	− 0.14	**1.00a**	0.03	0.10	− 0.42	0.07	0.46
Acidic flavor	− 0.14	− 0.26	− 0.25	− 0.13	− 0.13	− 0.21	0.24	0.21	0.20	0.24	0.01
Chockolate taste	**0.64a**	**0.78a**	− 0.43	− 0.08	− 0.30	0.40	− 0.52	− 0.52	0.21	− 0.12	**0.79a**
Cocoa taste	**0.76a**	**0.71a**	0.02	0.31	0.01	**0.58a**	− **0.59a**	− 0.56	0.15	0.00	**0.80a**
Sweetness	− 0.46	− 0.10	− 0.36	− **0.62a**	− 0.11	− **0.67a**	− 0.42	− 0.43	**0.67a**	0.07	− 0.18
Bitterness	**0.77a**	0.41	0.41	**0.73a**	0.01	**0.95a**	0.08	0.13	− 0.57	− 0.02	0.43
Astringency	**0.64a**	0.22	0.53	**0.79a**	0.11	**0.88a**	0.22	0.27	− **0.66a**	0.02	0.26
Acidic taste	**0.86a**	**0.67a**	0.10	0.50	− 0.12	**0.89a**	− 0.20	− 0.15	− 0.37	− 0.13	**0.67a**
2,3-Butanediol	**0.96a**	**0.70a**	0.16	0.56	0.01	**0.79a**	− 0.22	− 0.17	− 0.04	0.19	**0.82a**
1-Hexanol, 2-ethyl	0.56	0.52	− 0.31	− 0.01	− 0.19	0.43	0.06	0.07	− 0.39	− 0.15	0.50
Nonanal	**0.69a**	0.57	− 0.38	0.02	− **0.60a**	**0.58a**	0.05	0.05	− 0.14	0.06	**0.59a**
Benzaldehyde	**0.94a**	**0.58a**	0.08	0.46	− 0.32	**0.73a**	0.10	0.16	0.15	0.46	**0.75a**
Acetic acid	1.00	**0.73a**	0.02	0.43	− 0.23	**0.79a**	− 0.10	− 0.05	0.02	0.26	**0.85a**
Pentanoic acid	**0.73a**	1.00	− 0.38	− 0.07	− 0.23	0.41	− 0.46	− 0.47	0.26	− 0.19	**0.87a**
Isopentanoic acid	0.02	− 0.38	1.00	**0.87a**	0.55	0.44	0.08	0.15	− 0.42	0.10	− 0.36
Propanoic acid, 2-methyl	0.43	− 0.07	**0.87a**	1.00	0.39	**0.76a**	0.11	0.20	− 0.45	0.19	0.04
Butanoic acid, 4-chloro	− 0.23	− 0.23	0.55	0.39	1.00	− 0.13	− 0.33	− 0.31	− 0.28	− 0.21	− 0.22
Hexanoic acid	**0.79a**	0.41	0.44	**0.76a**	− 0.13	1.00	0.04	0.11	− 0.42	0.07	0.43
Propanoic acid, 2-methyl-, 3-hydroxy-2,4,4-trimethylpentyl ester	− 0.10	− 0.46	0.08	0.11	− 0.33	0.04	1.00	**0.99a**	− 0.26	0.43	− 0.36
Propanoic acid, 2-methyl-,2,2-dimethyl-1-(2-hydroxy-1-methylethyl) propyl ester	− 0.05	− 0.47	0.15	0.20	− 0.31	0.11	**0.99a**	1.00	− 0.27	0.49	− 0.36
2-Furancarboxaldehyde	0.02	0.26	− 0.42	− 0.45	− 0.28	− 0.42	− 0.26	− 0.27	1.00	0.37	0.33
2(3H)-Furanone, dihydro	0.26	− 0.19	0.10	0.19	− 0.21	0.07	0.43	0.49	0.37	1.00	0.08
2-Furanmethanol	**0.85a**	**0.87a**	− 0.36	0.04	− 0.22	0.43	− 0.36	− 0.36	0.33	0.08	1.00

The statistical data with letter “a”—were bolded and means statistically significant correlation between volatiles compounds and sensory attributes

**Fig. 5:**
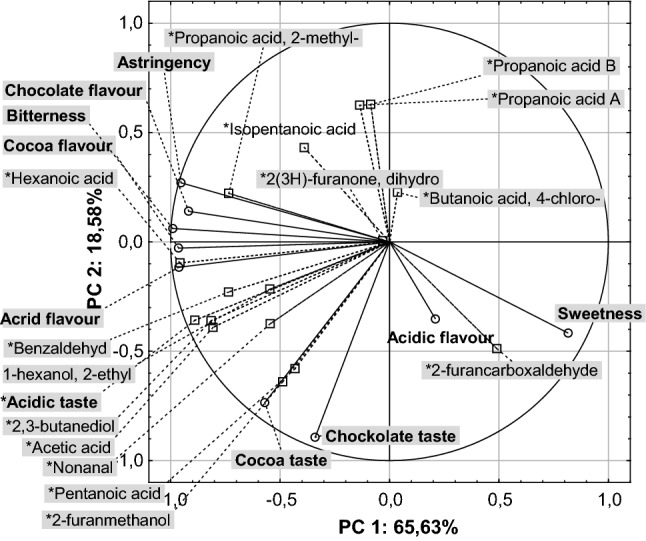
Principal component analysis of the volatile compounds and sensory attributes of the cocoa powder, mixtures and agglomerates of the powdered cocoa beverages, where: propanoic acid A means—propanoic acid, 2-methyl-, 3-hydroxy-2,4,4-trimethylpentyl ester, propanoic acid B—propanoic acid, 2-methyl-,2,2-dimethyl-1-(2-hydroxy-1-methylethyl) propyl ester and cyclohexanone…—cyclohexanone, 5-methyl-2-(1-methylethyl)-, (2R-cis)

PCA analysis (Fig. 5) was performed to correlate the volatile compounds with the results obtained from the sensory analysis. The data of volatile compounds were plotted on the basis of the relative percentages of individual compounds calculated from the total contents of volatiles in the chromatograms.

The PC1 (sensory discriminants) and PC2 (volatile compounds in cocoa products) explained about 65 and about 18% of the total variance (Fig. 5). The dominant compounds in all mixtures and agglomerates of cocoa beverages with additives were 2,3-butanediol, nonanal and acetic acid (PC2). In most of the analysed samples, there were esters: propanoic acid and furancarboxaldehyde. The remaining volatile compounds from different groups were present in some of the samples or in contents below the adopted level (Figs. 1, 2, 3).

On the positive part of PC1 axis (Fig. 5), a correlation was found between sweetness and acid flavour and 2-furancarboxaldehyde. According to Ribeiro et al. (2009), furancarboxaldehyde is characterised as spicy, sweet and with slightly caramel flavour and aroma, which confirms the correlation with sweetness. Acid notes can be due to the presence of acids, mainly acetic acid, in the cocoa powder, which strongly affects the consumer’s perceptions. The negative axis of PC1 focused mainly on cocoa and chocolate tastes, and both of them were correlated with compounds from different groups such as acetic acid, benzaldehyde, 1-hexanol, 2-ethyl or nonanal. Acetic acid with an acidic and acetic aroma is considered to be the compound with the most intense aroma in fermented and unroasted beans, while cocoa powder (P1; Table 1) is characterised by an intense and unacceptable acid taste and acrid flavour (Fig. 4). Cocoa beverages obtained from all the analysed mixtures (P6, P7, P8, P9, P10; Table 1) and their agglomerates (A6, A7, A8, A9, A10; Table 1) were accepted. Members of the sensory panel pointed to about a 5-times lower note in terms of these differentiators compared to cocoa powder (Fig. 4). In addition, the scores given by the panellists indicated lesser acid taste and acidic flavour sensations in the agglomerated samples, which confirms the contents of volatile compounds from the group of acids determined in the analysed products (Fig. 2).

According to Páramo et al. (2010) and Rodriguez-Campos et al. (2012), the high temperature used during drying or roasting decreases acids content in the product, while alcohols impart a fruity, fresh or floral aroma (Aprotosoaie et al. 2016). A high alcohol content is desirable to obtain cocoa products with floral and sweet notes (Rodriguez-Campos et al. 2012). The same authors also studied the effect of aldehydes and ketones on the sensory attributes of cocoa. They showed that aldehyde carbonyl compounds were crucial for the development of a good cocoa flavour (cocoa quality) (Rodriguez-Campos et al. 2012). In addition, they confirmed the effect of high temperature and longer roasting on aldehyde content reduction. The presence of nonanal or benzaldehyde was correlated with the cocoa taste and chocolate taste, and therefore the distinguishing features expected in processed cocoa bean products confirm the correlation between volatile compounds from the group of aldehydes and acids and sensory attributes characteristic of cocoa products.

A strong correlation (Fig. 5) was demonstrated between acrid flavour and hexanoic acid content, which can result from undesirable, bitter and sweet pungent flavour of this compound. Acetic acid imparting the acidic taste to cocoa products; undesirable 2,3-butanediol, pungent benzaldehyde causing bitter or fruity taste; and herbal 1-hexanol, 2-etyl showed a strong correlation with cocoa taste and chocolate flavour (Fig. 5). In addition, pentanoic acid, which is assigned undesirable features, as well as sweet and honey, was also in the negative PC1 field. These compounds create the characteristic cocoa and chocolate notes. Results of our study confirm findings reported by Ramli et al. (2006), Rodriguez-Campos et al. (2012), and Aprotosoaie et al. (2016).

A correlation was also found between acids (hexanoic, propanoic acid, 2-methyl and isopentanoic) and flavour (chocolate, cocoa, astringency and bitterness). The bitterness and tartness characteristic of cocoa beans are also perceptible in the processed products, including cocoa powder. In addition, the chocolate, floral, biscuit flavour is attributed to hexanoic acid, hence may be correlated with the flavourings. On the other hand, propanoic acid can cause negative and undesirable qualities, including rancid taste (Aprotosoaie et al. 2016).

Undesirable and pungent features are associated with the presence of hexanoic acid, believed to be a compound that imparts unpleasant taste and aroma to chocolate products (da Veiga Moreira et al. 2016). These authors showed a correlation between astringency, bitterness, acrid flavour, and content of propanoic acid. Pentanoic acid, 2-methyl and hexanoic acid were found in cocoa powder. However, the percentage of cocoa powder in the mixtures at the level of 20% in relation to the total weight of the mixtures resulted in the less noticeable features resulting from the presence of these compounds. Therefore, sensory analysis pointed out the notes of astringency, bitterness, acrid flavour, chocolate and cocoa flavour, which, however, were not overcome by negative impressions.

There was no positive correlation between volatile compounds and sensory attributes on axis PC2. The relationships between two different groups i.e. butanoic acid,4-chloro from acid group and 2(3H)-furanone, dihydro belonging to the furanone group did not correlate with any sensory characteristics of the analysed products.

The first compound was present only in the sample A10 (Table 1), whereas the other in the agglomerated cocoa powder (A1; Table 1), in the mixture with basic composition (A10; Table 1), and in the mixture P10 (Table 1) (Fig. 5).

## Conclusion

The volatile compounds of the studied powders and powdered cocoa beverages belonged in particular to six various groups such as alcohols, acids and esters, aldehydes and ketones, and cyclic components including furans. 2,3-Butanediol was present in cocoa powder (it was in the highest concentration), its mixtures and agglomerates. Acetic acid which dominated in the group of acids, was found in 13 of the 20 tested samples, and its highest content was determined in cocoa powder. No clear effect of modification of the raw material composition on content of the determined acid was observed. Nonanal was a compound present in all tested samples. Cyclic components were found in some samples, mainly mixtures, and as a result of agglomeration they were reduced below 0.5%. In the majority of the tested powdered cocoa beverages, the agglomeration caused a decrease in contents of dominating volatile compounds, compared to a mixture of the same composition.

The greatest differences were determined for acetic acid and 2,3-butanediol. The statistical inference showed the influence of the agglomeration process on the profile of volatile compounds and the impact of modification of the raw material composition on the sensory characteristics. A correlation was found between volatile compounds content and specific sensory variables. The cocoa mixture characteristics as well as processing conditions affect contents of volatile compounds and sensory quality of the powdered cocoa beverages.
